# High expression of BTN3A1 is associated with clinical and immunological characteristics and predicts a poor prognosis in advanced human gliomas

**DOI:** 10.3389/fimmu.2024.1397486

**Published:** 2024-05-28

**Authors:** Abdou-samad Kone, Amina Ghouzlani, Ahmed Qandouci, Nour el Imane Issam Salah, Yann Bakoukou, Abdelhakim Lakhdar, Mehdi Karkouri, Abdallah Badou

**Affiliations:** ^1^ Immuno-Genetics and Human Pathology Laboratory (LIGEP), Faculty of Medicine and Pharmacy, Hassan II University, Casablanca, Morocco; ^2^ Department of Neurosurgery, University Hospital Center (UHC) Ibn Rochd, Casablanca, Morocco; ^3^ Laboratory of Pathological Anatomy, University Hospital Center (CHU) Ibn Rochd, Hassan II University, Casablanca, Morocco; ^4^ Mohammed VI Center for Research and Innovation, Rabat, Morocco and Mohammed VI University of Sciences and Health, Casablanca, Morocco

**Keywords:** BTN3A1, gliomas, immune checkpoint, immunotherapy, prognostic factor

## Abstract

**Introduction:**

Gliomas represent the most prevalent and aggressive tumors within the central nervous system. Despite the current standard treatments, the median survival time for glioblastoma patients remains dismal, hovering around 14 months. While attempts have been made to inhibit the PD-1/PD-L1 and CTLA-4/CD80-CD86 axes through immunotherapy, the outcomes have yet to demonstrate significant efficacy. The immune checkpoint Butyrophilin 3A1 (BTN3A1) can either be involved in advantageous or detrimental function depending on the cancer type.

**Methods:**

In our study, we utilized a Moroccan cohort to delve into the role of BTN3A1 in gliomas. A transcriptomic analysis was conducted on 34 patients, which was then corroborated through a protein analysis in 27 patients and validated using the TCGA database (n = 667).

**Results:**

Our results revealed an elevated expression of BTN3A1 in glioblastoma (grade 4), as evidenced in both the TCGA database and our cohort of Moroccan glioma patients. Within the TCGA cohort, BTN3A1 expression was notably higher in patients with wild-type IDH. We observed a positive correlation between BTN3A1 expression and immune infiltration of B cells, CD8+ T cells, naive CD4+ T cells, and M2 macrophages. Patients exhibiting increased BTN3A1 expression also presented elevated levels of TGF‐β, IL‐10, and TIM‐3 compared to those with reduced BTN3A1 expression. Notably, patients with high BTN3A1 expression were associated with a poorer prognosis than their counterparts with lower expression.

**Conclussion:**

Our findings suggest that BTN3A1 might promote the establishment of an immunosuppressive microenvironment. Consequently, targeting BTN3A1 could offer novel therapeutic avenues for the management of advanced gliomas.

## Introduction

Gliomas are malignant brain tumors that originate from glial cells within the Central Nervous System (CNS) and are notorious for their rapid growth and invasiveness (1). Glioblastoma (GBM) stands out as the most prevalent and lethal subtype, accounting for approximately 57% of all gliomas and 48% of primary CNS malignant tumors. Patients with GBM face a median survival rate of merely 14–16 months (1, 2). Over the past decade, there has been a significant refinement in the World Health Organization’s classification of gliomas. The 5th edition of the WHO classification of Tumors of the Central Nervous System (WHO CNS5) has incorporated specific molecular and genetic criteria. As a result, GBMs are now delineated as grade 4 gliomas with a wild-type IDH (3). Furthermore, the classification distinguishes various histological types: adult-diffuse gliomas, pediatric low-grade diffuse gliomas, pediatric high-grade diffuse gliomas, and circumscribed astrocytic gliomas (4). Specific mutations further categorize adult diffuse gliomas into astrocytoma (with mutated IDH), oligodendroglioma (featuring mutated IDH and a 1p/19q code), and glioblastoma (with wild-type IDH) (4). It is crucial to highlight that the terms “pediatric” and “adult” types in this context do not refer to the patient’s age but rather to distinct molecular and genetic profiles (5).

This paradigmatic transition underscores the need to re-evaluate both diagnostic and therapeutic strategies. The emphasis is now on personalized medicine, particularly in the realm of immunotherapy. The CNS, contrary to previous beliefs, is an immuno-competent region (6). However, the immunosuppressive microenvironment in GBM tumors, fueled by a combination of molecular and cellular elements such as interleukin-6 (IL-6), interleukin-10 (IL-10), transforming growth factor-beta (TGF-β), M2 macrophages, microglia, and regulatory T cells (T-regs), thwarts effective anti-tumor responses (7).

In this context, numerous studies have underscored the significance of examining the role of immune checkpoint (IC) molecules and the potential benefits of inhibiting these pathways (8–13). To date, the Food and Drug Administration (FDA) has approved two immune checkpoint blockade (ICB) therapeutic strategies (14). Anti-PD1 and/or anti-CTLA-4 drugs are commonly administered to patients with advanced and recurrent glioblastoma and may be combined with temozolomide (TMZ)-based chemotherapy or radiotherapy (15, 16). While these therapies offer certain benefits, such as reinvigorating the functional state of tumor-infiltrating lymphocytes (TILs) and enhancing the expression of Interferon-Gamma (IFN-γ) and Interleukin-2 (IL-2), they have yet to demonstrate a significant improvement in overall survival (16, 17). Therefore, it might be prudent to investigate novel blockade approaches, focusing on promising immune checkpoints like VISTA, TIGIT, A2AR, and NR2F6, all of which have been linked to advanced glioma (18–21).

Consistent with this approach, the present study seeks to ascertain the potential role of Butyrophilin Subfamily 3 Member A1 (BTN3A1 or CD277) in advanced gliomas. BTN3A molecules are members of the immunoglobulin family and exist in three distinct isoforms: BTN3A1, BTN3A2, and BTN3A3, which exhibit 95% of structural homology (22). These paralogous genes encode transmembrane proteins featuring both IgV and IgC extracellular domains, as well as a B30.2 intracellular region pivotal for downstream signaling (23). BTN3A1 appears to be the only one that can efficiently elicit signal transduction, either after stimulation by binding to its putative ligand on its extracellular domain, or by the direct interaction of a phosphoantigen with the B30.2 intracellular domain. Studies have highlighted the significant role of the intracellular domain B30.2 in cell signaling (24). Unlike BTN3A1 and BTN3A3, BTN3A2 lacks it, which limits its involvement in signaling processes (24). Regarding BTN3A3, despite the presence of the intracellular domain B30.2, it would appear to be less efficient than BTN3A1 in cell signaling due to slight changes in amino acid residues in the B30.2 structure (25) The BTN3A1 molecule is expressed on a variety of cells, including T cells, B cells, monocytes, dendritic cells, natural killer (NK) cells, and even tumor cells (26). Most studies on BTN3A1 focus mainly on how it activates γδ T cells, whereas BTN3A molecules are also involved in αβ T cell immunomodulation (27). As previously demonstrated, BTN3A1 is implicated in CD4+/CD8+ αβ T-cells inhibition. Using genetically engineered APCs expressing BTN3A1, Kyle K. Payne et al. demonstrated reduced IFNγ release and CD4+/CD8+ T-cell proliferation (27). Jinghua. W and coworkers highlighted the interaction between BTN3A1 on T cells and LSECtin on melanoma cells, resulting in decreased production of IFN-γ, IL-2, and TNF-α (28). The ability of BTN3A2 and BTN3A3 to induce these inhibitions on CD4+/CD8+ αβ T-cells has not yet been fully demonstrated. While some studies have identified LSECtin as a ligand for BTN3A1, the definitive binding partner for this molecule remains inadequately characterized (28, 29). As such, the prognostic value of BTN3A1 is intricately tied to the specific cancer type and the signaling pathways activated upon binding with its presumptive ligand on APC/tumor cells (26). For instance, overexpression of BTN3A1 in ovarian cancer (OC) has been linked to suppressed αβ T cell proliferation and reduced T helper 1 (Th1) cytokine production, culminating in unfavorable patient outcomes (30). Additionally, the correlation of BTN3A expression with immune evasion strategies of pancreatic tumor (PC) cells reinforces the role of BTN3A in facilitating tumor progression (31). A notable recent study elucidated that BTN3A1 overexpression in esophageal squamous cell carcinoma (ESCC) augments radioresistance by modulating the expression and phosphorylation of UNC-51-like autophagy-activating kinase (ULK1), subsequently correlating with adverse prognoses (32).

In our study, we endeavored to probe and underscore the significance of BTN3A1 in gliomas. By leveraging the TCGA database in tandem with our in-house cohort, we scrutinized the relationship between BTN3A1 expression, and clinicopathological as well as molecular factors. Our findings indicated that BTN3A1 could play a part in promoting tumor progression, and its overexpression correlates with poor prognosis. We are optimistic that our findings will pave the way for devising potential anti-BTN3A1 therapeutic avenues for addressing advanced gliomas.

## Materials and methods

### Ethics approval and consent to participate

The present study received approval from the Ethical Board of Ibn Rochd University Hospital, Casablanca, with the approval code 28/15. Written informed consent was secured from all glioma patients, with consent from parents or legal guardians obtained for participants below 18 years of age. All methods were conducted in accordance with relevant guidelines and regulations.

### Patients and specimens

A total of 34 biopsies were included in our transcriptomic analysis: 9 specimens of grade 1, 7 of grade 2, 7 of grade 3, and 11 of grade 4/Glioblastoma (refer to **Supplementary Material 1** for clinicopathological parameters). Additionally, 27 formalin-fixed and paraffin-embedded (FFPE) glioma tissues were assessed, consisting of 6 specimens of grade 1, 5 of grade 2, 8 of grade 3, and 8 of grade 4/Glioblastoma. All samples were sourced from patients who underwent neurosurgical resection of gliomas at the Neurosurgery Department of Ibn Rochd University Hospital. Every participant was informed about the research purpose, and written consent was obtained.

### Data source and data processing

We procured patient mRNA expression data and associated clinical information from the Merged Cohort (Lower Grade Glioma/TCGA, Firehose Legacy + Glioblastoma Multiforme/TCGA, Firehose Legacy) via the open access database at http://cbioportal.org. Any samples lacking RNA-seq data or clinicopathological details were excluded and our inclusion criteria encompassed comprehensive RNA-seq data and clinicopathological details for each specimen. A total of n = 667 patients was then taken into account for downstream analyses. (refer to **Supplementary Material 2** for data processing).

### Normalization methods

During the analysis, initial normalization was conducted using the DESeq2 function available at https://bioconductor.org/packages/release/bioc/. Subsequently, RNAseq expression values underwent Log2 transformation to ensure dependable results for differential gene expression and comparisons among samples. We delineated the “high BTN3A1” and “low BTN3A1” clusters based on the median. These designations respectively represent patients with elevated and reduced BTN3A1 expression.

### RNA extraction, complementary DNA synthesis

Total mRNA was isolated and purified from fresh frozen tissues (n=34) employing the Trizol reagent (Invitrogen, France) as described by the manufacturer’s protocol. We gauged the quality and concentration of the extracted RNA with the NanoVue™ Plus spectrophotometer (GE Healthcare, UK). The cDNA synthesis was performed in two steps. Initially, approximately 1 μg of total RNA was mixed with 1 μl of Random Hexamer Primer (25 μg, Bioline, France) and 4 μl of RNase-Free Water. This mixture was incubated at 70°C for 5 minutes, serving to dismantle the RNA’s secondary structure and ready the reaction milieu. Subsequently, a solution containing 4 μl of Tetro Reverse Transcriptase buffer, 4 μl of dNTP (10 mM), 0.5 μl of RNase Inhibitor (Invitrogen, France), 0.5 μl Tetro Reverse Transcriptase Enzyme (Bioline, France), and 1 μl of RNase-Free Water was added. This was incubated at 25°C for 10 minutes, 45°C for 30 minutes, and finally at 85°C for 5 minutes.

### Quantitative real-time polymerase chain reaction

For qRT-PCR, we employed the SYBR™ Green PCR Master Mix (Thermo Fischer), adhering to the manufacturer’s specifications, and utilized the croBEE® Real-Time PCR Detection System. The ubiquitously expressed β-Actin gene served as an internal benchmark, facilitating the assessment of BTN3A1’s relative expression. The PCR was programmed as follows: an initial step of 10 minutes at 95°C for polymerase activation and sample denaturation. This was succeeded by 40 cycles of 15 seconds at 95°C for cDNA double-strand denaturation and 1 minute at 60°C for primer hybridization and extension, and finally annealing at 95°C for 15 seconds, 60°C for 1 minute and 95°C for 15 seconds. This final phase is imperative for overseeing amplification quality and specificity. The mRNA expression was measured using the 2^-ΔCt (ΔCt = Ct target gene − Ct β-Actin) method. The Sequences of the primers for qPCR are designed as:

BTN3A1 Forward Primer: 5’-CTTCAGCTGCTCATGCCTCA-3’
BTN3A1 Reverse Primer: 5’-CAGATCAGCGTCTTCACCCA-3’
β-Actin Forward Primer: 5′- GAGATGGCCACGGCTGCTT-3′
β-Actin Reverse Primer: 5′- GCCACAGGACTCCATGCCCA-3′

### Immunohistochemical detection of BTN3A1

Formalin-fixed paraffin-embedded (FFPE) glioma tissue blocks, comprising 6 specimens of grade 1, 5 of grade 2, 8 of grade 3, and 8 of grade 4 (Glioblastoma), were sectioned into 4-μm thickness. These sections were then mounted on slides using an albumin-water solution and dried in an oven at 60°C for one hour and subsequently stored at 37°C overnight. The immunohistochemical staining was conducted using the Dako EnVision™ FLEX, High pH (Link) (Code K8000) detection system. Antigen retrieval employed the Heat-Induced Epitope Retrieval (HIER) method in a Tris/EDTA buffer (pH 9), subjected to 98°C for 20 minutes. To inhibit endogenous peroxidase activity, slides were treated with the EnVision FLEX peroxidase blocking reagent (Dako, Denmark) for 10 minutes at ambient temperature. After which, they were rinsed twice with the wash buffer (EnVision flex wash buffer, Dako) for 2 minutes each. Primary antibodies, BTN3A1 polyclonal antibody (OACA02656) (https://www.avivasysbio.com/btn3a1-antibody-oaca02656.html) at a 1:300 dilution and rabbit IgG isotype control (bs-0295P) (https://www.biossusa.com/products/bs-0295p) at a 1:200 dilution, were applied to the tissue sections and allowed to incubate for 30 minutes at room temperature. Post incubation, slides were washed as described and then treated with a horseradish peroxidase-conjugated goat anti-rabbit IgG secondary antibody (EnVision Flex/HRP, Dako, USA) for 20 minutes at room temperature. DAB chromogen was subsequently applied for 10 minutes, highlighting the binding of the BTN3A1 antibody to the tissue. Counterstaining was achieved using Hematoxylin and eosin for one minute, followed by sequential dehydration in ethanol and toluene baths. Slides were then mounted for observation under a light microscope.

### Staining quantification

Membrane and cytoplasmic expression of BTN3A1 in glioma tissues was evaluated to provide a semi-quantitative measure of protein expression. This was determined using the Immunoreactivity Score (IRS), which ranges from 0 to 12. The IRS is calculated as follows: IRS = Percentage of Positive Cell Staining (PS) (ranging from 0 to 4) multiplied by Staining Intensity (SI) (ranging from 0 to 3).

For the Percentage of Positive Cell Staining:

• 0 corresponds to 0%
• 1 corresponds to 1–24%
• 2 corresponds to 25–49%
• 3 corresponds to 50–74%
• 4 corresponds to 75–100%

For Staining Intensity:

• 0 denotes Absence
• 1 denotes Low
• 2 denotes Moderate
• 3 denotes Strong

### Estimation of tumor-infiltrating immune cells by TIMER

The Tumor IMmune Estimation Resource (TIMER) (http://timer.cistrome.org/) facilitates a comprehensive analysis and estimation of tumor-infiltrating immune cells. In the TCGA GBM cohort (n=153), we assessed the correlation between BTN3A1 expression and the immune infiltration of CD8+ T cells, CD4+ T cells, B cells, and macrophages. A Spearman correlation was considered significant at p < 0.05.

### Analysis of single-cell expression of BTN3A1 by TISCH

BTN3A1 expression at the single-cell level across various cell types was analyzed using the GSE databases: Glioma_GSE131928_Smartseq2 (https://www.ncbi.nlm.nih.gov/geo/query/acc.cgi?acc=GSE131928) (33) and Glioma_GSE163108_Smartseq2 (https://www.ncbi.nlm.nih.gov/geo/query/acc.cgi?acc=GSE163108) (34) both of which are available on the Tumor Immune Single-cell Hub (TISCH) open-access database (http://tisch.comp-genomics.org).

### Enrichment analysis

We conducted the gene set enrichment analysis using the GSEA v4.3.2 software (https://www.gsea-msigdb.org/gsea/downloads.jsp). Based on the median value, patients were categorized into High-BTN3A1” and “Low-BTN3A1” groups in glioblastoma. We then evaluated the association between BTN3A1 expression and hallmark genes from biological immune pathways. Nominal p values were estimated using 1000 permutations. Results were considered statistically significant if the p value was less than 0.05 and the False Discovery Rate (FDR) was below 0.25.

### Statistical analysis

All figures and statistical analyses were executed using GraphPad Prism v8.0.1. We applied the non-parametric Mann-Whitney test for comparisons between two independent conditions/groups and the Kruskal-Wallis test when comparing gene expression across more than two conditions/groups. The correlation between selected genes was determined using Spearman’s non-parametric test. All statistical tests were two-tailed, with significance set at p < 0.05. For survival differences between groups, the Kaplan-Meier survival curve analysis, based on the log-rank test, was employed. Two independent individuals in the laboratory assessed the various statistical tests.

## Results

### BTN3A1 is upregulated in glioblastoma/Grade 4 and IDH wild-type glioma

**Figure 1:**
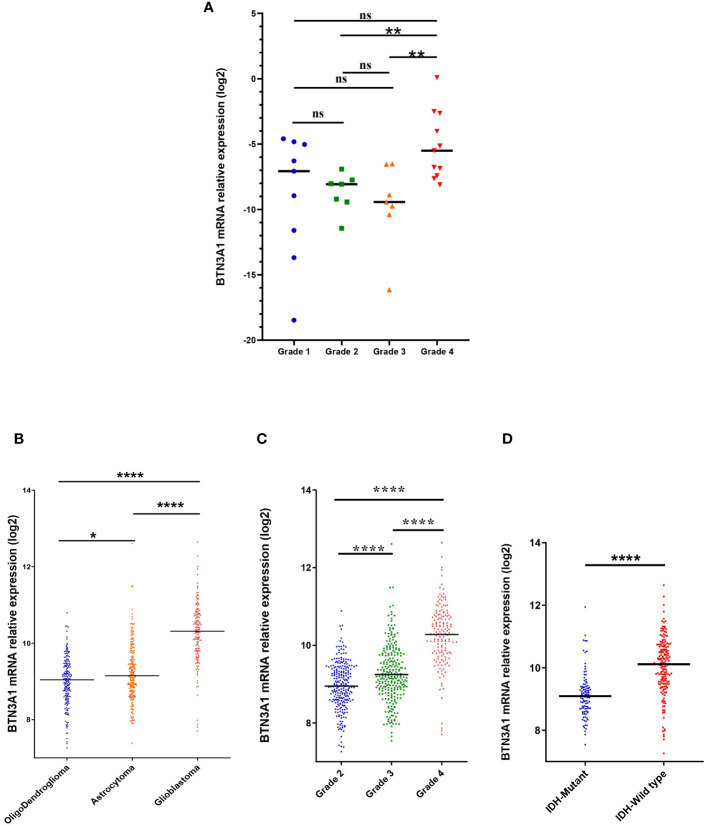
Association between BTN3A1 expression and clinical parameters. **(A)** High level of BTN3A1 mRNA expression in grade 4 Moroccan glioma patients (n = 34). **(B)** BTN3A1 was upregulated in Glioblastoma compared with Oligodendroglioma and Astrocytoma in TCGA dataset. **(C)** BTN3A1 expression is associated with grade progression in TCGA dataset. **(D)** IDH-Wild-Type glioma patients exhibit high levels of BTN3A mRNA in TCGA dataset. ns, not significant; *p-value < 0.05; **p-value < 0.01; ***p-value < 0.001; ****p-value < 0.0001.

We first evaluated BTN3A1 mRNA expression in our cohort of Moroccan glioma patients (n = 34): 9 patients with grade 1, 7 with grade 2, 7 with grade 3, and 11 with grade 4/Glioblastoma. We found that BTN3A1 expression was higher in Grade 4 compared to both Grade 2 (p < 0.01; **Figure 1A**) and Grade 3 (p < 0.01; **Figure 1A**>). Our findings were further validated in the TCGA cohort. We assessed BTN3A1 transcriptomic expression according to various clinicopathological parameters (**Table 1**; **Figures 1B–D**), considering the 5th edition of the WHO classification of central nervous system tumors (4). In the TCGA dataset, BTN3A1 expression was significantly higher in Glioblastoma compared to both Oligodendroglioma and Astrocytoma (p < 0.0001; **Figure 1B**). This higher expression in grade 4 was also seen when compared to grades 3 and 2 (p < 0.0001; **Figure 1C**). Regarding the IDH status, IDH wild-type patients exhibited elevated BTN3A1 expression compared to their IDH mutant counterparts (p < 0.0001; **Figure 1D**).

**Table 1:** Expression of BTN3A1 according to the clinicopathological parameters in the glioma patient of the TCGA dataset.

Parameters	Number (%)	P-value
Sex		
Male	381 (57%)	0,4083
Female	285 (43%)	
WHO grade (5th edition)		
Low grade (2)	249 (37,4%)	
High grade (3–4)	417 (62,6%)	<0,0001
Histological type		
Astrocytoma (IDH-mutant)	194 (36%)	
Oligodendroglioma (IDH-mutant and 1p/19q-codeleted)	191 (36%)	
Glioblastoma (IDH-wild-type)	152 (28%)	<0,0001
IDH mutation status		
Mutant	385 (72%)	
Wild-type	152 (28%)	<0,0001

**Figure 2:**
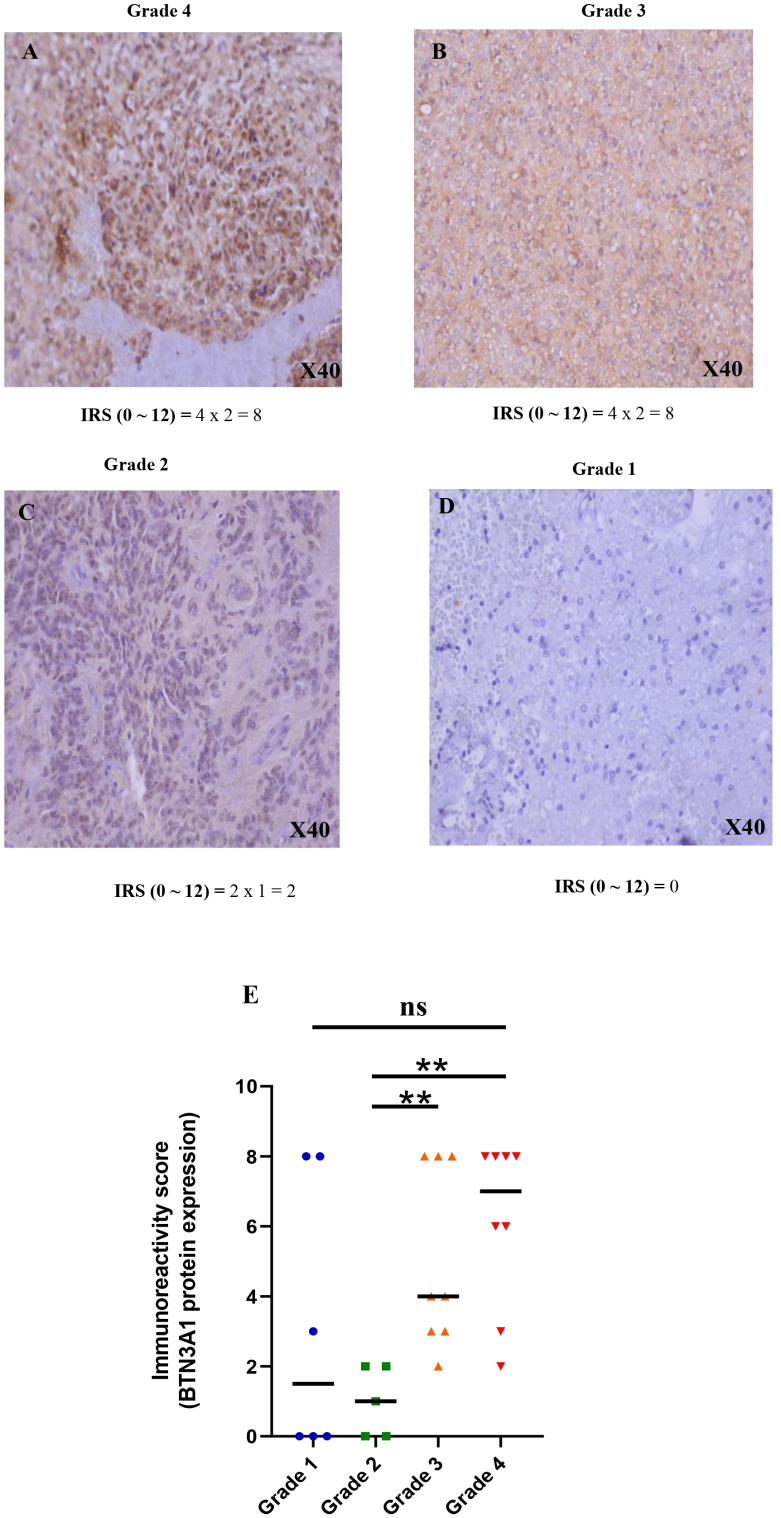
Immunohistochemical staining of BTN3A1 using anti-BTN3A1 (OACA02656) and 3,30-diaminobenzidine (DAB; brown). Haematoxylin was used for nuclear counterstaining (blue). **(A)** Strong positive staining with anti BTN3A1 in grade 4 glioma tissue (magnification ×40). **(B)** Strong positive staining with anti BTN3A1 in grade 3 glioma tissue (magnification ×40). **(C)** Low positive staining with anti BTN3A1 in grade 2 glioma tissue (magnification ×40). **(D)** BTN3A1-negative staining with anti BTN3A1 in grade 1 glioma tissue. **(E)** Representation of the difference in BTN3A1 expression between the different glioma grades. The Immunoreactivity-score IRS (0 ~ 12) = Percentage of positive cell staining (PS) (0 ~ 4) x Staining Intensity (SI) (0 ~ 3). Scale bare 100 µm; ns, not significant; *p-value < 0.05; **p-value < 0.01; ***p-value < 0.001; ****p-value < 0.0001.

To complement our transcriptomic data, we examined the protein expression profile of BTN3A1 by immunohistochemistry, using samples from 6 patients with grade 1, 5 with grade 2, 8 with grade 3, and 8 with grade 4/Glioblastoma. In alignment with our earlier results, BTN3A1 was significantly upregulated in grade 4 and grade 3 gliomas compared to grade 2 (p < 0.01; **Figure 2**).

In summary, BTN3A1 expression appeared associated to a more aggressive and pathogenic state of glioma.

### BTN3A1 expression, detected in both immune and tumor cells, correlates with the infiltration of B cells, CD8+ T cells, CD4+ T cells (Th1), Treg, and Macrophages (M0, M2) in glioblastoma

**Figure 3:**
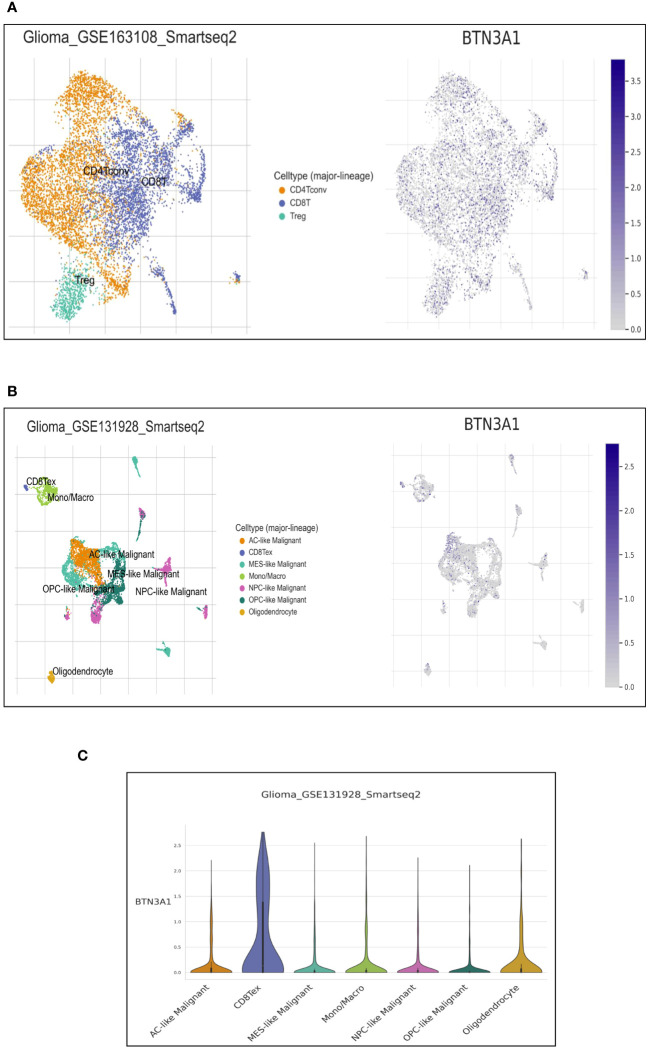
Single-cell expression of BTN3A1 in immune and malignant cells in Glioblastoma. **(A, B)**, Uniform Manifold Approximation and Projection (UMAP) plot showing the distribution and expression levels of BTN3A1 in different immune and malignant cell types in the GSE163108_Smartseq2 and GSE131928_Smarseq2 datasets. **(C)** The distribution of BTN3A1 expression levels in the GSE131928_Smarseq2 dataset. AC-like, Astrocyte-Like Malignant; MES-like, Mesenchymal-like; Mono/Macro, monocyte-macrophages; NPC, neural-progenitor; OPC, oligodendrocyte-progenitor.

The immune infiltration profile within the tumor microenvironment critically informs the characterization of the immune phenotype. Highlighting the correlation between BTN3A1 expression and immune cell type infiltration can offer deeper insights into the immune system’s modulation. Utilizing the Tumor Immune Single Cell Hub (TISCH) web resource—specifically the GSE163108_Smartseq2 (https://www.ncbi.nlm.nih.gov/geo/query/acc.cgi?acc=GSE163108) andGSE131928_Smarseq2 (https://www.ncbi.nlm.nih.gov/geo/query/acc.cgi?acc=GSE131928) databases— we found BTN3A1 expressed at the single-cell level in conventional CD4+ T cells, CD8+ T cells, and CD4+ Treg cells (**Figure 3A**). Notably, BTN3A1 expression was observed in exhausted CD8 +T cells and pro-tumor cells, including astrocyte-like (AC-like) cells (**Figures 3B, C**).

**Figure 4:**
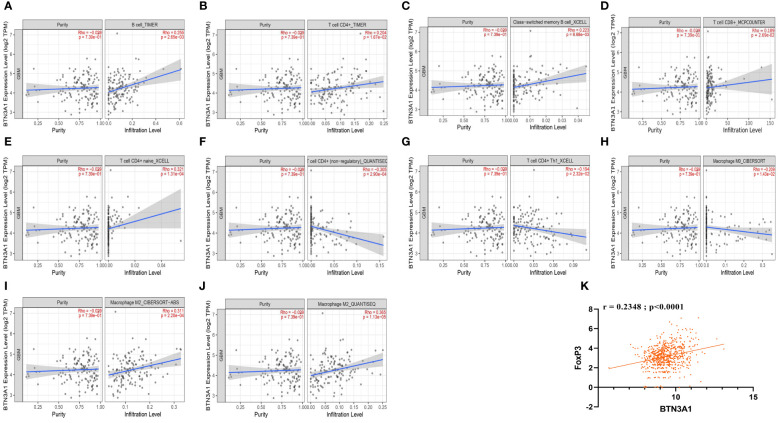
The correlation between BTN3A1 expression and infiltration of B cells, CD4+ T cells, CD8+ T cells, and Macrophages in Glioblastoma. **(A, B)** Estimation of B cell and CD4+ T cell infiltration using the Timer algorithm. **(C, E, G)**, Estimation of the infiltration of memory B cells, naive CD4+ T cells and Th1 CD4+ T cells using the XCELL algorithm. **(D)** Estimation of CD8+ T cell infiltration using the MCPCOUNTER algorithm. **(F, J)** Estimation of non-regulatory CD4+ T cell, M2 macrophage infiltration using QUANTISEC algorithm.**(H, I)** Estimation of M0 and M2 macrophage infiltration using the CIBERSORT algorithm. TIMER, quanTIseq and CIBERSORT algorithms use deconvolution-based methods to estimate immune cell infiltration. xCell and MCP-counter algorithms use the Marker-based approaches to estimate Immune cell Infiltration. **(K)** BTN3A1 expression is positively correlated with Tregs (FoxP3) in our Glioblastoma TCGA database.

We further probed the correlation between BTN3A1 expression and the infiltration of these specific immune cells using the Tumor IMmune Estimation Resource (TIMER). For more reliable results, we employed the two principal methodologies for estimating immune infiltration in the tumor microenvironment: the deconvolution-based method (via TIMER, MCP-Counter, CIBERSORT) and the marker-based approach (xCell, quanTIseq). Interestingly, TIMER estimations revealed positive correlations between BTN3A1 expression and the immune infiltration of B cells and CD4+ T cells (**Figures 4A, B**). Moreover, BTN3A1 expression correlated positively with immune infiltration of memory B cells, CD8+ T cells, and naive CD4+ T cells as gauged by the xCell and MCP-Counter methods (**Figures 4C–E**).

In accordance with the TISCH analysis, BTN3A1 expression is associated with pro-tumor immune characteristics. We identified a negative correlation between BTN3A1 expression and M0 macrophages, non-regulatory CD4+ T cells especially Th1 cells (**Figures 4F–H**. Conversely, a positive correlation was observed with M2 macrophage immune infiltration as measured by CIBERSORT and quanTIseq (**Figures 4I, J**). Additionally, our TCGA data indicated a positive correlation between Treg (FoxP3) and BTN3A1 expression (**Figure 4K**; r = 0.2348; p < 0.0001).

Taken together, our findings suggest that BTN3A1 might foster an immunosuppressive phenotype within the Glioblastoma patient’s microenvironment.

### Highly expressed BTN3A1 is associated with pro-tumour immune-related molecules in glioblastoma

**Figure 5:**
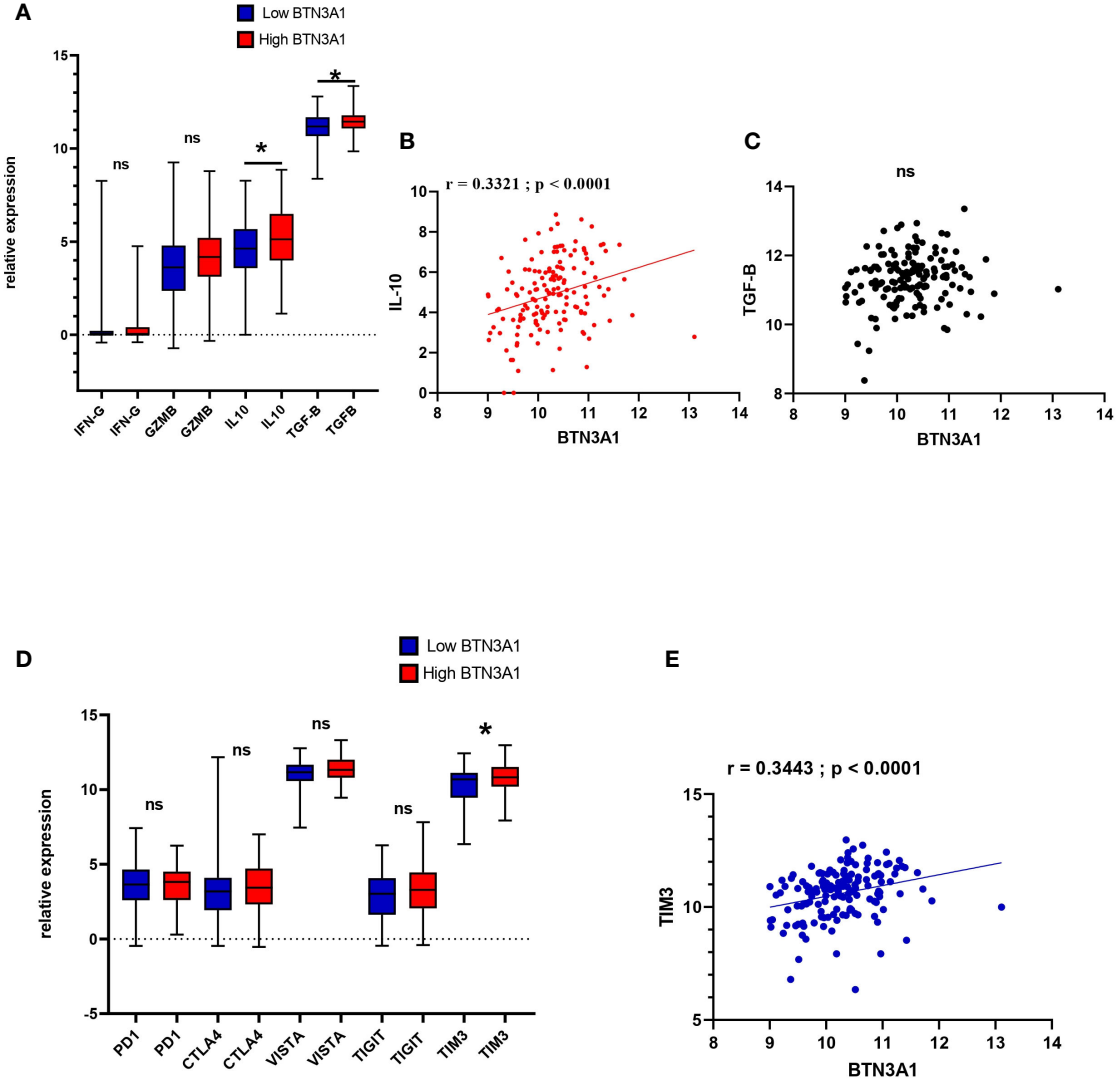
Expression profile of anti and pro-tumor immune-related molecules according to Low and High BTN3A1 expression in glioblastoma **(A)** Higher expression of BTN3A1 is associated with a slight increase in IL-10 and TGF-β levels. **(B)** BTN3A1 expression is positively correlated with IL-10 expression. **(C)** No correlation between BTN3A1 expression and TGF-β. **(D)** Higher BTN3A1 expression is associated with high levels of TIM3. **(E)** Positive correlation between BTN3A1 expression and TIM3. ns, not significant; *p-value < 0.05; **p-value < 0.01; ***p-value < 0.001; ****p-value < 0.0001

The nature of the immune response in the tumour microenvironment is shaped by a myriad of interactions among cells and factors, including cytokines, chemokines, cytoplasmic granule toxins, and both soluble and membrane-bound immune checkpoints (35). Building upon our previous results into immune cell infiltration and its relationship with BTN3A1 expression, we delved deeper into the association between BTN3A1 expression and both pro- and anti-tumor molecules. Our results revealed that a slight increase in the expression of IL-10 and TGF-β is associated with heightened levels of BTN3A1 (**Figure 5A**; p-value < 0.05). Specifically, there was a positive correlation between BTN3A1 expression and IL-10 (**Figure 5B**) (r = 0.3321; p < 0.0001). However, no significant correlation was identified between BTN3A1 expression and TGF-β (**Figure 5C**). With regard to inhibitory immune checkpoints, only TIM3 expression was notably higher in patients with elevated BTN3A1 expression compared to those with reduced BTN3A1 levels (**Figure 5D**; p-value < 0.05). This observation is further reinforced by the significant positive correlation observed between BTN3A1 and TIM3 expression (**Figure 5E**) (r = 0.3443; p < 0.0001).

It is plausible, therefore, to hypothesize that BTN3A1 may facilitate immunosuppression in the tumor microenvironment in conjunction with TIM3. This hypothesis will be elaborated upon and critically assessed in the discussion section.

### Association between elevated BTN3A1 expression and immune pathway-related hallmark genes in glioblastoma

**Figure 6:**
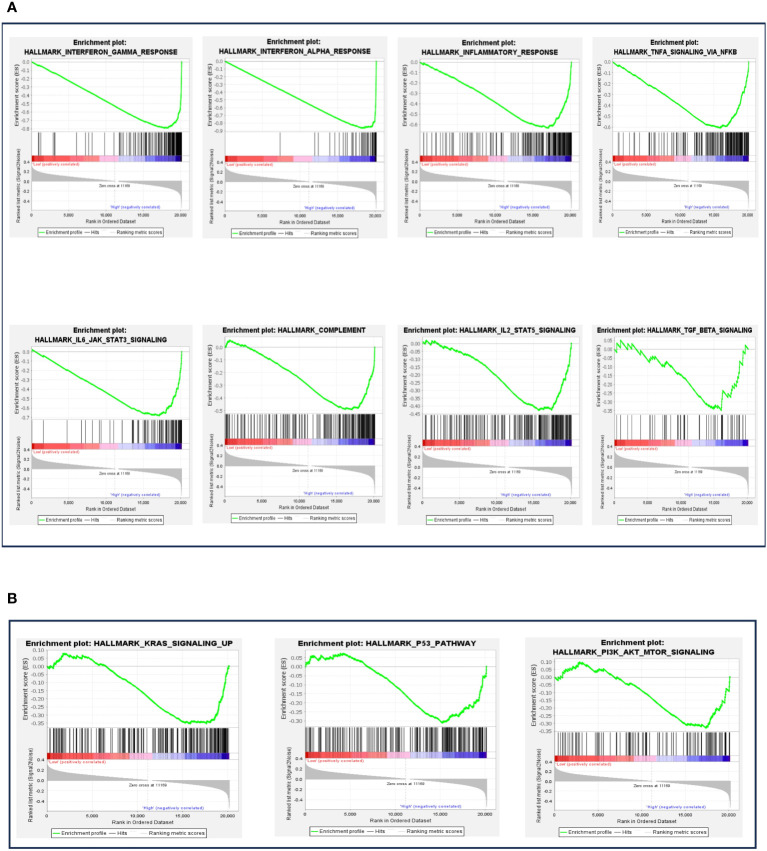
Gene set enrichment analysis (GSEA) according to BTN3A1 mRNA expression. **(A)** The different plots represent the significant immune pathways-related hallmark genes in the “high-BTN3A1” group. **(B)** The different plots represent the significant protumoral pathways-related hallmark genes in the “high-BTN3A1” group. The ranking metric measures a gene’s correlation with a phenotype. The value of the ranking metric goes from positive to negative as you move down the ranked list. A positive value indicates correlation with the first phenotype (Low-BTN3A1 group) and a negative value indicates correlation with the second phenotype (High BTN3A1 group).

In our analysis, the next logical step was to evaluate the various biological and immune pathways linked to BTN3A1 expression. We employed the DEseq method to normalize RNAseq data, and based on the median expression, patients were categorized into two groups: “high-BTN3A1” and “low-BTN3A1”.”. A Gene Set Enrichment Analysis (GSEA) was subsequently conducted to pinpoint hallmark genes associated with immune processes. We deemed results as statistically significant if the p-value was below 0.05 and the false discovery rate (FDR) did not exceed 0.25. In the “high-BTN3A1” group, 35 pathways were enriched, of which 20 were statistically significant (refer to **Supplementary Material 3**). Eight of these 20 pathways were enriched in hallmark genes associated with the immune response (HALLMARK_INTERFERON_GAMMA_RESPONSE) (HALLMARK_INTERFERON_ALPHA_RESPONSE) (HALLMARK_INFLAMMATORY RESPONSE) (HALLMARK_TNFA_SIGNALING_VIA_NFKB) (HALLMARK_IL6_JAK_STAT3_SIGNALING) (HALLMARK_COMPLEMENT) (HALLMARK_IL2_STAT5_SIGNALING) (HALLMARK_TGF_BETA_SIGNALING) (**Figure 6A**) and three pathways were linked to tumor progression (HALLMARK_KRAS_SIGNALING_UP) (HALLMARK_P53_PATHWAY) (HALLMARK_PI3K_AKT_MTOR_SIGNALING) (**Figure 6B**). On the other hand, no significant immune or tumour processes were observed in the “low BTN3A1” group, except for the HALLMARK_MYC_TARGETS_V1 and HALLMARK_MYC_TARGETS_V2 pathways (**Supplementary Material 4**).

These findings indicate that BTN3A1 expression may play a role in modulating inflammatory responses and potentially promote the activation of tumorigenic processes in glioblastoma.

### BTN3A1 upregulation is associated with poor outcomes in human glioma patients

In light of our previous observations, we evaluated the influence of BTN3A1 expression on patient survival. We conducted a Kaplan-Meier survival analysis comparing patients with low BTN3A1 expression to those with high BTN3A1 expression in the TCGA dataset. The analysis included all patients for whom expression, event and month data were available. Of the 667 patients, only 665 and 629 had complete information for the OS and DFS analyses respectively. Missing information was marked as NA: Not Available in the TCGA clinical data and was therefore not considered.

**Figure 7:**
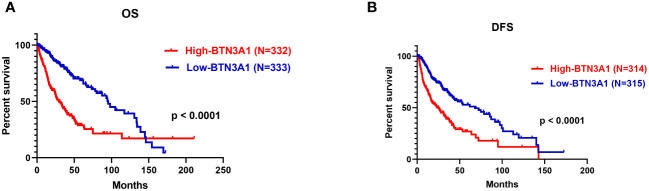
Survival analysis according to BTN3A1 expression in all glioma patients in the TCGA dataset. **(A)** Higher BTN3A1 expression is associated with worse Overall Survival (OS) in glioma patients. **(B)** Higher BTN3A1 expression is associated with worse Disease-Free Survival (DFS) in glioma patients.

We observed that upregulation of BTN3A1 was associated with a significant decrease in both OS (**Figure 7A**; p < 0.0001) and DFS (**Figure 7B**; p < 0.0001).

From these findings, we could infer that high levels of BTN3A1 adversely affect the survival of patients diagnosed with high-grade gliomas.

## Discussion

The central nervous system is an intricate and meticulously organized structure responsible for many crucial biological functions. Pathological disturbances within this system can lead to significant lifestyle implications (36). Glioblastoma (GBM), which accounts for over 50% of gliomas, is classified as an IDH-wildtype and grade 4 tumor according to the latest WHO classification of the CNS (4). The aggressive and invasive nature of GBM can compromise the integrity of the blood-brain barrier (BBB) and hampers the CNS’s proper functioning, resulting in a markedly low survival rate (37). Immunotherapy emerges as a promising therapeutic strategy that extends patient care beyond conventional treatments (38). While CTLA-4 and PD-1/PD-L1 immune checkpoint blockades have yielded significant results in various cancers, such as melanoma, non-small cell lung cancer (NSCLC), and bladder cancer (BC), their survival benefits have not yet been established in gliomas (39–41). Consequently, the exploration of new immune checkpoint candidate molecules might yield better outcomes for glioma patients. The BTN3A1 (CD277) molecule, a member of the immunoglobulin superfamily receptors, has been shown to play a role in Vγ9Vδ2 T-cell activation through the sensitivity of its B30.2 domain to phosphoantigens (pAg). Recently, there has been growing interest in investigating the relationship between BTN3A1 and cancer, with prior results indicating its dual role in tumor progression (26). In this study, we assessed the expression pattern and potential role of BTN3A1 using both TCGA datasets and our own Moroccan glioma cohort.

Transcriptomic and protein expression analyses of BTN3A1 expression in our Moroccan cohort revealed a significant upregulation in grade 4 gliomas when compared to grades 2 and 3. This was corroborated by the TCGA dataset, which displayed a heightened expression of BTN3A1 in grade 4 gliomas relative to grades 2 and 3, and also in grade 3 compared to grade 2. Following the World Health Organization CNS5 guidelines, glioblastomas are now categorized as IDH-WILD type/grade 4 gliomas (5). Our findings suggest that an increased expression of BTN3A1 is associated with more aggressive glioma subtypes. This suggests that BTN3A1 could serve as a potential biomarker for glioma diagnosis and grading. In clinical scenarios, elevated BTN3A1 expression could raise suspicion for grade 4 gliomas, leading to a more precise classification and facilitating timely and appropriate clinical management. Previous research has demonstrated that soluble plasma levels of BTN3A1 (sBTN3A1) may predict the efficacy of nivolumab therapy in patients with metastatic renal cell carcinoma (mRCC) (42). Therefore, recognizing elevated BTN3A1 expression as a marker for grade 4 gliomas could augment the molecular characteristics delineated in the fifth edition of the WHO classification of central nervous system tumors (WHO CNS5).

Our single-cell analysis indicated that BTN3A1 is expressed in both immune and glioma cells. Interestingly, Recent findings have delineated four distinct glioblastoma cell states: Neural-Progenitor-like (NPC-like), Oligodendrocyte-Progenitor-like (OPC-like), Astrocyte-like (AC-like), and Mesenchymal-like (MES-like). Each of these states mirrors the neurodevelopmental differentiation and damage responses observed in the normal neural lineage (33). While the reaction of normal neural cells to tumor cells plays a role in shaping the tumor microenvironment, altered responses from glioblastoma progenitor cells lead to overlapping effects that significantly influence the propagation and persistence of glioblastoma (33). Our results revealed an upregulation of BTN3A1 in the AC-like cell state. Notably, the AC-Like state is characterized by aggressive and proliferative cells (33). Furthermore, adult diffuse gliomas exhibit a higher proportion of AC-Like cells compared to pediatric diffuse gliomas. This distinction could partially account for the more aggressive nature of adult gliomas (33, 43). Given these findings, the pronounced expression of BTN3A1 in AC-Like cells supports our initial observations, underscoring BTN3A1’s potential as a promising marker for glioblastomas.

With respect to the profile of immune infiltration, we observed that BTN3A1 expression directly correlated with the infiltration levels of B cells, CD8+ T cells, general CD4+ T cell populations, and M2 macrophages. Conversely, there was a negative correlation with Th1 and M0 macrophages. Recent research posits the Central Nervous System (CNS) as an immune-competent region. Moreover, evidence indicates that the glioma environment can impair and render the Blood-Brain Barrier (BBB) permeable, thus promoting the migration of immune effectors (6, 44).

In light of our findings, BTN3A1 seems to drive a pro-tumor immune response by bolstering M2 macrophage activities. Studies have demonstrated that M2 macrophages foster tumor progression and engender an immunosuppressive microenvironment within gliomas, notably through the secretion of cytokines IL-6, TGF-β, and IL-10, which in turn recruit Treg cells (45, 46). This assertion aligns seamlessly with our observations where elevated BTN3A1 levels were associated with heightened IL-10 and TGF-β expression. However, there wasn’t any significant difference in the expression of IFN-γ and Granzyme B between “high-BTN3A1” and “low-BTN3A1” groups. Notably, BTN3A1 exhibited a positive correlation with IL-10 and FoxP3, and single-cell analysis indicated that exhausted CD8+ T cells expressed BTN3A1.

Drawing from the data, we hypothesize that (1) BTN3A1 propels its pro-tumor effect in glioblastoma by inciting M2 macrophages to produce IL-10. This, in turn, activates and attracts Treg cells, culminating in CD8+ T-cell exhaustion. Earlier research also identified an association between BTN3A1 upregulation in ovarian cancer and the suppression of αβ T cell activity, ostensibly due to BTN3A1’s interference with CD45-mediated signaling, thereby obstructing TCR-mediated T cell activation (27). Adding to this, Wang J. et al. presented evidence that BTN3A1, when expressed on T-cells, binds to LSECtin on melanoma cells, resulting in the suppression of T cell proliferation and the production of cytokines like (28). A holistic analysis spanning various omics data sets revealed a positive association between BTN3A1 and immune cells such as B cells, CD8+ T cells, CD4+ T cells, macrophages, neutrophils, and dendritic cells in conditions like Breast Cancer (BRCA), Lung Adenocarcinoma (LUAD), and Lung Squamous Cell Carcinoma (LUSC) (47).

Our research underscored the presence of BTN3A1 in both immune and glioblastoma cells. Its expression was clearly associated with immune cell infiltration and immunosuppressive activity. We highlighted the potential role of BTN3A1 in the establishment of an immunosuppressive microenvironment, thus contributing to the inhibition of αβ T cell activity in gliomas. In a recent study, Wang Y and coworkers demonstrated that high BTN3A1 protein expression could predict a favorable Vγ9Vδ2 T-cell-mediated cytotoxic response in glioblastoma (48). Increased levels of BTN3A1 may enhance phosphoantigen presentation to γδ T cells, improving their anti-tumor responses. In such a glioma microenvironment, the higher the expression of BTN3A1, the more effective the cytotoxicity of γδ T cells should be, leading to tumour regression and improved prognosis. Although this hypothesis is fully cogent, it does, have some limitations. γδ T cells are uncommon, representing less than 5% of all T lymphocytes (49). In addition, as mentioned in the related study, the cytotoxicity of the γδ T cells against GBM cells was limited (≈30%) (48). High levels of BTN3A1 will tip the balance in favor of tumour progression and αβ T cell inhibition, despite the low compensation of γδ T cell activation. By prioritizing the reversal of αβ T cell inhibition over γδ T cell activation, our results suggest that blocking BTN3A1 may be an effective strategy and will represent a promising avenue for immunotherapy in glioblastoma. Alternatively, a strategy based on preventing BTN3A1-induced inhibition of αβ T cells, combined with stimulation of γδ T cells, will contribute meaningfully to improving anti-tumor response. In their study, Kyle K. Payne et al. demonstrated that CTX-2026, an anti-BTN3A1 antibody, can both hinder BTN3A1-mediated inhibition of αβ T-cells and activate γδ T cells (27). To summarize, the tumor microenvironment specificity is pivotal and governs how some therapeutic strategies can be implemented to foster relevant outcomes. Our findings suggest targeting BTN3A1 may present a promising avenue for immunotherapy in glioblastoma.

Combined therapies have consistently demonstrated superior efficacy compared to monotherapies. Notably, while the efficacy of immunotherapies for glioblastoma remains constrained, there’s compelling evidence supporting enhanced survival outcomes through combination immune checkpoint blockade strategies (50). In our investigation into the relationship between BTN3A1 and various checkpoints such as PD-1, CTLA-4, VISTA, TIGIT, and TIM-3, we discerned a significant association. Elevated BTN3A1 expression correlated positively with heightened TIM-3 expression in glioblastoma. Previous research has highlighted TIM-3’s role in advancing glioblastoma progression by curtailing the effector functions of CD8+ T cells and Th1 cells and attenuating IFNγ production (51). Additionally, TIM-3 plays a pivotal role in the migration and polarization of pro-tumoral M2 macrophages (52). Our findings, juxtaposed with prior research, suggest that (2) BTN3A1 might expedite glioma progression through the activation of the IL-6/JAK/STAT3 pathway (as depicted in **Figure 6A**). This leads to the upregulation of TIM-3, potentially inhibiting T cell activities while stimulating M2 macrophages. Furthermore, existing studies have characterized a pro-tumoral glioma phenotype distinctively marked by the TIM-3/interleukin 6 interaction (51, 52). It should also be noted that in addition to the IL-6/JAK/STAT3 pathway, Tim-3 seems to promote tumor progression through the NF-κB signaling pathway (53). Herein, we found that high expression of BTN3A1 was associated with the activation of the NF-κB via the TNF-α signaling pathway. The involvement of NF-κB in oncogenesis is well established (54, 55) Thus, co-expression of BTN3A1 with TIM-3 may result in a synergistic pro-tumor effect by enhancing the IL-6/JAK/STAT3 and TNF-α/NF-κB pathways. Previous studies demonstrated that the combination of anti-TIM-3 with anti-PD1 has aroused more encouraging results than with anti-PDL1 in melanoma, NSCLC, and ESCC (53). Combined immune checkpoint blockade is a fairly recent strategy in the treatment of gliomas, as evidenced by the ongoing clinical trial which will assess the combination of Anti-Tim-3 with Anti-PD-1 (56). This positive correlation between BTN3A1 and TIM-3 holds promising implications for potential combined immune-checkpoint blockade strategies in glioblastoma.

Our GSEA analysis probed the influence of BTN3A1 in orchestrating both immunological and tumor pathways. Overexpression of BTN3A1 was associated with hallmark genes implicated in inflammatory responses, encompassing INF-α, INF-γ, TNF-α, and the complement system. Distinct research underscores the ambivalent role of these inflammatory agents in glioblastoma (57). For instance, in addition to INF-α, INF-γ, and TNF-α’s pro-inflammatory functions, INF-γ has been shown to amplify PD-L1 expression and foster T-reg differentiation in glioblastoma, thereby abetting glioma cells in evading immune surveillance (7, 58). Preliminary transcriptomic analyses aimed at deriving a risk score grounded on INF-γ expression posited that elevated INF-γ levels correlated with a more adverse clinicopathological and molecular prognosis in glioblastoma (59, 60). Simultaneously, TNF-α exhibited heightened expression in GBM and was implicated in bolstering tumor proliferation (61). Current literature remains scant regarding the roles of INF-α and the complement system in glioblastoma. Our research suggests that BTN3A1 is potentially instrumental in triggering inflammatory cascades, as evidenced by its association with hallmark genes of inflammatory responses. Subsequently, an immune-suppressive microenvironment seems to be established, steered by increased expression of BTN3A1. Furthermore, our data reveals an association of heightened BTN3A1 expression in glioblastoma with signature genes of pathways such as IL6/JAK/STAT3, TGF-β, KRAS, P53, and PI3K/AKT/MTOR. It’s intriguing that BTN3A1’s role in tumorigenesis can oscillate between pro-tumoral and anti-tumoral, contingent on the pathological milieu and the specific downstream signaling cascades activated by specific interactions between the IgC extracellular domain of BTN3A1 and its putative ligand (26, 29). Prior investigations affirm the profound implications of pathways like KRAS, P53, and PI3K/AKT/MTOR in the onset, evolution, and metastasis of glioblastoma (62–64). We postulate that (3) BTN3A1 may champion glioblastoma establishment and expansion by actuating the KRAS, P53, and PI3K/AKT/MTOR signaling conduits. BTN3A1’s correlation with hallmark genes of the IL6/JAK/STAT3 and TGF-β pathways reaffirms and validates our preceding observations. These observations indicated that (1) BTN3A1 mediates a pro-tumoral influence in glioblastoma, principally through the induction of M2 macrophages to synthesize IL-10 and TGF-β. This cascade eventually leads to CD8+ T-cell exhaustion and (2) accentuates glioma development via the IL-6/JAK/STAT3 pathway.

The analysis of BTN3A1’s prognostic significance revealed an association between elevated BTN3A1 expression and poorer prognosis in glioma patients. Elevated BTN3A1 expression has also been linked to unfavorable outcomes in esophageal squamous cell carcinoma (ESCC), metastatic gastrointestinal stromal tumors (mGISTs), and ovarian cancer (OC) (27, 32, 65).

The present study suggests that BTN3A1 expression can serve as a prognostic indicator for worse outcomes in advanced human gliomas.

## Conclusion

In summary, our study posits that elevated BTN3A1 expression correlates with an immunosuppressive microenvironment in gliomas, precipitating a heightened aggressive pathological state. BTN3A1 seems to foster a pro-tumor response, operating in association with TIM3, M2 macrophages, Tregs, and other pro-tumor pathways. Consequently, BTN3A1 expression may serve as a pioneering immunological indicator to foresee unfavorable outcomes in advanced human gliomas.

However, our study remains limited due to the limited size of our Moroccan patient cohort. Further exploration is essential to validate the mechanistic and functional significance of BTN3A1 in the modulation of immune cells, paving the way for potential anti-BTN3A1 immunotherapies.

## Data availability statement

The original contributions presented in the study are included in the article/**Supplementary Materials**, further inquiries can be directed to the corresponding author/s.

## Ethics statement

The present study received approval from the Ethical Board of Ibn Rochd University Hospital, Casablanca, with the approval code 28/15. The studies were conducted in accordance with the local legislation and institutional requirements. Written informed consent for participation in this study was provided by the participants’ legal guardians/next of kin.

## Author contributions

AK: Conceptualization, Data curation, Formal analysis, Investigation, Methodology, Software, Supervision, Validation, Visualization, Writing – original draft, Writing – review & editing. AG: Data curation, Formal analysis, Visualization, Writing – review & editing. AQ: Data curation, Formal analysis, Software, Writing – review & editing. NS: Formal analysis, Investigation, Writing – review & editing. YB: Formal analysis, Writing – review & editing. AL: Data curation, Writing – review & editing. MK: Data curation, Formal analysis, Writing – review & editing. AB: Funding acquisition, Methodology, Supervision, Validation, Visualization, Writing – review & editing.
