# Supplementary material for: High expression of BTN3A1 is associated with clinical and immunological characteristics and predicts a poor prognosis in advanced human gliomas
Source: Front Immunol. 2024 May 28;15:1397486. doi: 10.3389/fimmu.2024.1397486 (PMC11165028; doi:10.3389/fimmu.2024.1397486)
Supplement: Supplementary file 1 [file Table_2.docx]

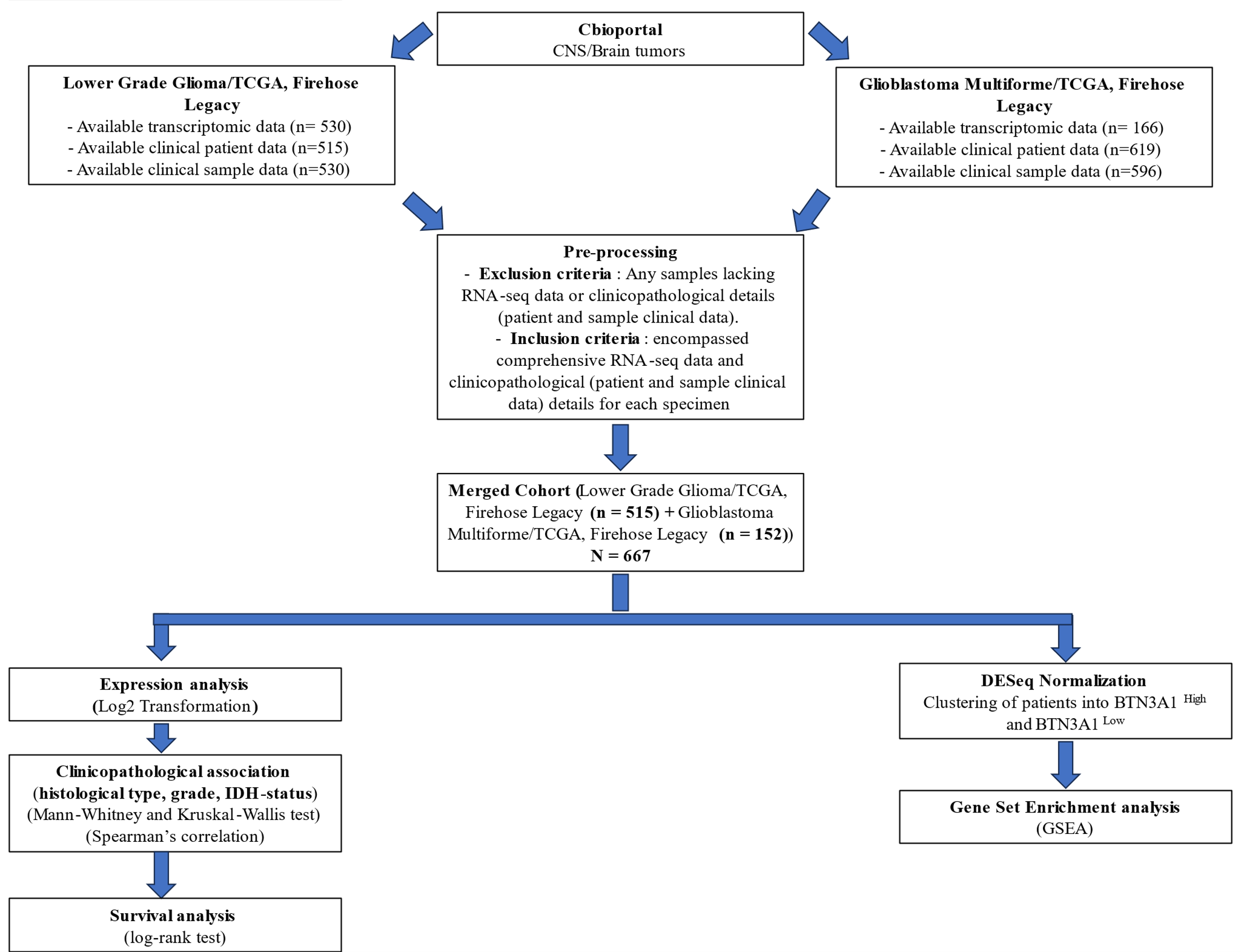


**Supplementary material 2**. The workflow for processing RNAseq data from the Cbioportal open-access online public database. Patients with available transcriptomic and clinicopathological data were prioritized in the pre-processing step.

The code for DESeq analysis is publicly available on [**https://github.com/Ackerman95/Dseq2-code-TCGA**](https://github.com/Ackerman95/Dseq2-code-TCGA)
